# Genetic Insights into the Historical Attribution of Variety Names of Sweet Chestnut (*Castanea sativa* Mill.) in Northern Italy

**DOI:** 10.3390/genes15070866

**Published:** 2024-07-01

**Authors:** Marta Cavallini, Gianluca Lombardo, Claudio Cantini, Mauro Gerosa, Giorgio Binelli

**Affiliations:** 1 Department of Biotechnology and Life Sciences (DBSV), University of Insubria, 21100 Varese, Italy; m.cavallini1@studenti.uninsubria.it (M.C.); gianluca.lombardo@uninsubria.it (G.L.); 2 Institute of Bioeconomy (IBE), Consiglio Nazionale Ricerche (CNR), 58022 Follonica, Italy; claudio.cantini@ibe.cnr.it; 3 Associazione Castanicoltori Lario Orientale, 23851 Sala al Barro, Italy; mauro@associazionecastanicoltorilarioorientale.it

**Keywords:** *Castanea sativa*, SSR, chestnuts, conservation, heirloom varieties, phylogeny, Bayesian analysis

## Abstract

The sweet chestnut (*Castanea sativa* Mill.) is subject to the progressive disappearance of its traditional chestnut groves. In the northern part of Italy, where distribution of the sweet chestnut is fragmented, many local varieties continue to be identified mostly by oral tradition. We characterised by SSRs eleven historically recognised varieties of sweet chestnut in the area surrounding Lake Como, with the goal of giving a genetic basis to the traditional classification. We performed classical analysis about differentiation and used Bayesian approaches to detect population structure and to reconstruct demography. The results revealed that historical and genetic classifications are loosely linked when chestnut fruits are just “castagne”, that is, normal fruits, but increasingly overlap where “marroni” (the most prized fruits) are concerned. Bayesian classification allowed us to identify a homogeneous gene cluster not recognised in the traditional assessment of the varieties and to reconstruct possible routes used for the propagation of sweet chestnut. We also reconstructed ancestral relationships between the different gene pools involved and dated ancestral lineages whose results fit with palynological data. We suggest that conservation strategies based on a genetic evaluation of the resource should also rely on traditional cultural heritage, which could reveal new sources of germplasm.

## 1. Introduction

Increased interest in biodiversity related problems has spotlighted previously unconsidered traditional aspects of the diversity of ecosystems, species diversity and genetic diversity. This is also happening for forest trees, where territory types are now being increasingly considered in the traditional, or “compositional”, assessments of diversity [1,2], as well as territorial models being considered in the component of biodiversity dealing with demographic and genetic structure, or “structural” biodiversity [3].

Another important issue arising is the revival of cultural heritage, not limited to cultural property, but in a broader view encompassing “natural heritage”, or, simply put, biodiversity. Thus, cultural heritage can be seen as not only “a way to manage the past for the future” on an abstract level but an important issue in the conservation of genetic resources.

The sweet edible chestnut (*Castanea sativa* Mill.) is a perfect species for which to put this issue into practice. Its very expansion and today’s distribution demonstrate this. In fact, the natural diffusion of sweet chestnut has been accompanied by an anthropogenic expansion that has strongly modified its initial range [4,5]. The discovery in the northwest of Turkey of fossil pollen dated to 18 kyr B.P. [6] and the reduction in the level of genetic diversity moving westward from Asia [7,8] indicates a millenary cultivation of chestnut trees and an anthropogenic expansion in Southern Europe more than a natural one, through varietal selection [7]. Asia Minor and the Transcaucasian region are to be considered the centre of domestication, cultivation and expansion of the chestnut tree [7,9].

In Italy, it is widespread in high hills and mountain environments, characterising some types of forests present in the phytoclimatic zone that takes its name, Castanetum (according to Pavari’s phytoclimatic classification [10]), extending from the plain to the submontane belt. Chestnut trees are an integral part of Italy’s natural and cultural landscape, and they have a significant distribution across the country, mainly in Campania, Lazio, Tuscany, Emilia-Romagna, Piedmont and Lombardy. The progressive disappearance of the traditional chestnut groves must be ascribed to many concomitant factors starting from difficult accessibility, multiple waves of pathogens and pests, socio-economic factors, such as the need for coppicing and tannin production, and urbanisation of the human population, among many others [9]. However, in the northern part of Italy, where distribution of sweet chestnut has been fragmented because of the orographic conditions, many varieties remain, many of which have been passed down from one family member to another over many generations, even centuries, as can be proved by looking at Middle-Age documents attesting to the existence of “premium” varieties [11]. These varieties fully deserve the definition of heirloom cultivars, not least because their reproduction by grafting has maintained the original characteristics.

The assessment of geographical patterns of genetic diversity and the identification of populations and areas harbouring high amounts of it has been explored in several papers, with special focus on Europe [12,13], and also to set conservation priorities. At a more local scale, genetic diversity has been studied in sweet chestnut, mainly referring to the traditional division between “chestnuts” and “marrons”, the latter being more sought after on the market for their organoleptic characteristics, among which are size, texture and flavour. Marron stands display a high degree of genetic homogeneity derived from constant human selection [14,15,16], which is a contrast to the high genetic diversity among chestnut cultivars [17,18]. Historically, marron stands have been prized and defined since the Middle Ages, and also formed part of tributes due to landlords; many Italian marron varieties are still named according to their geographic origin, even though genetic characterisation of Italian marrons possibly indicates a common origin [15].

To the best of our knowledge, no efforts have been made to ascertain the concordance of history and genetics for sweet chestnut. This work involves the genetic characterisation of eleven grafted and historically recognised varieties of sweet chestnut in a region of Northern Italy near Lake Como (known historically as Lario), chosen from among those regions with the most trees present, with the main goal of giving a genetic basis to traditional varieties that have been historically defined by oral tradition. Two approaches were used: the first is based on a Bayesian reconstruction of sweet chestnut phylogeny to provide insights into how historical events such as glaciation shaped the current biogeographical patterns of the species, the second is based on a traditional assessment of genetic diversity and differentiation among the varieties, to focus on more recent events that have acted at the regional scale. This will allow us to build germplasm collections representative of a broad range of phylogenetic diversity as a regional conservation strategy and the results are to be used in final product characterisation, food exploitation, multifunctional strategies of Castanetum and propagation programs.

## 2. Materials and Methods

### 2.1. Plant Material, DNA Extraction and Amplification

To assess the genetic identity of the sweet chestnuts in the Lario region, samples belonging to eleven identified historical varieties were collected (Table S1), from a total of 368 trees. The sampling was performed on the basis of the local given names for each variety: the term “Marrone” indicates presumed marrons, the root “marron” refers, instead, to marrons-like varieties and all the other varieties are considered sweet chestnuts. Samples were retrieved with the help of local chestnut growers from five different mountain communities in Lombardy: Lario Orientale e Valle San Martino, Valsassina, Triangolo Lariano, Valchiavenna and Valli del Lario e del Ceresio (Figure S1). All sampled trees were grafts, with the exception of the Bianchit variety.

Leaves were sampled in the upper part of the crown, in all cases above the grafting point, to ensure that the material belonged to the grafted cultivar (original scion) and not to the rootstock. The sampling was carried out in two ways. First, we sampled green, juvenile and healthy leaves, free from phytosanitary problems. These were stored in ice for transport and then stored at −20 °C. We then tried to use dried leaves to ease sampling during the autumn season.

Genomic DNA was extracted by grinding ~100 mg of leaf in a mortar with liquid nitrogen with a small amount of sand to increase abrasion. Lysis buffer from the DNeasy Plant Mini Kit (Qiagen, Hilden, Germany) was then added together with 20 µL of proteinase K (20 mg/mL) and four sterile steel beads. This was vortexed for 15–20 min until the mixture was smooth and rid of leaf pieces. This was left at 56 °C overnight. The successive steps were carried out following manufacturer’s instructions.

Standard end-point polymerase chain reactions (PCR) were performed on a Mastercycler Gradient (Eppendorf^®^ AG, Hamburg, Germany) to amplify DNA with the following conditions: an initial step of 5 min at 95 °C, followed by 30 cycles of 30 s at 95 °C, 90 s at the annealing temperature of each primer pair, 30 s at 72 °C and with a final extension step of 30 min at 60 °C. A set of 13 homologous markers, chosen based on their polymorphism, were selected: CsCAT1, CsCAT2, CsCAT3, CsCAT6, CsCAT14, CsCAT16, CsCAT17, CsCAT34 and CsCAT41 [19] and EMCs22, EMCs25, EMCs32 and EMCs38 [20]. Forward primers were labelled with different fluorophores (6-FAM, HEX, ROX and TAMRA) for multiplexed genotyping (Table S2). All PCR reactions were conducted in a final volume of 25 µL, containing 5 µL of 5x MyTaq™ reaction buffer (5 mM dNTPs and 15 mM MgCl2); 0.3 µM of each forward and reverse primer; 0.5 µL of MyTaq™ DNA polymerase (Meridian Bioscience Inc. Cincinnati, OH, USA) and ~40–60 ng of template DNA. The PCR products were sent to be genotyped externally (Macrogen, Seul, Republic of Korea). To score the genotype of each sample, the microsatellites profiles were checked and analysed using Peak Scanner v.2.0 Fragment Analysis software (ThermoFisher Scientific, Waltham, MA, USA).

### 2.2. Data Analysis

Analyses were based on well-established population genetics principles in order to estimate basic descriptive statistics. First, to assess the genetic variability among the eleven varieties, the mean number of alleles per locus (Na), observed and expected heterozygosity (*H_o_* and *H_e_*) was obtained using the software Genetix v.4.05.2 [21]. Genetix was then used to evaluate the level of population differentiation using pairwise *F*_ST_ statistics among populations using Weir and Cockerham’s estimator [22], and to assess the degree of inbreeding by calculating *F*_IS_. The deviation of Wright’s *F*-statistics from zero was tested for all loci in all individuals under the null hypothesis of Hardy–Weinberg equilibrium by 1000 permutations. A pairwise Nei’s distances matrix [23] was calculated with 1000 repetitions between populations and results were displayed with MegaX v.11.0.13 (Tamura et al., Tokyo, Japan; Philadelphia, PA, USA). A multivariate analysis, PCoA (Principal Coordinate Analysis), based on pairwise genetic distances between individuals was performed using Genetix and plotted using the “ggplot2” [24] and “scatterplot3d” v.0.3-44 [25] R packages.

The mean number of effective alleles per locus (Nea), mean number of private alleles per locus (Npa) and the percentage of polymorphic loci (% pl) were estimated using GenAlEx v.6.51b2 [26].

In order to examine the presence of genetic structuring in the studied populations, we used a Bayesian clustering analysis method implemented in the software Structure v.2.3.1 [27]. Firstly, we determined the most likely number of ancestral gene pools, *K*, based on all microsatellite genotypes. We ran 20 independent tests for each *K* value ranging from 1 to 15. Tests were performed using a “no admixture” model [28], 100,000 MCMC iterations and a 10,000-iteration burn-in period. We then used *structureHarvester* v.0.6.94 [29] with the following arguments: -*-evanno* --*clumpp*. This calculates the most likely number of ancestral gene pools using *ΔK* method [30] and averages each single *K* runs into separate files. Individual runs for each *K* value were then summarised using crimp v.1.1.0 [31] with the following arguments, *-n* 20 (number of runs) and *-r* (output aligned coefficient matrices). Finally, output from crimp was used to generate the genetic structure bar plot using StructuRly v.0.1.0 and outputting by population and *Q*-values [32].

**Figure 1:**
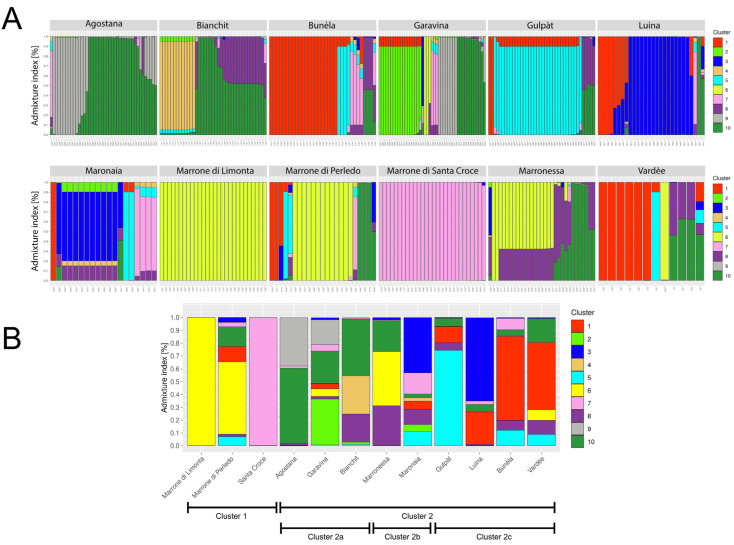
(**A**) Genetic structure bar plot of the studied samples as inferred by Bayesian clustering for *K* = 10. Each vertical bar represents a single individual and its proportion of membership to a genetic pool is indicated by the different colours. (**B**) Average population structure for each variety as inferred by Bayesian clustering for *K* = 10. Each vertical bar represents a single variety and its proportion of membership to a genetic pool is indicated by the different colours.

Demographic analyses were performed using Beast v2.7.6 [33]. Genepop allele data were converted into fstat format and loaded in using the Beast2 package beastvntr v.0.2.0 [34]. Site model was set using 8 γ categories with its shape distribution estimated and substitution model in tree branches was calculated using the Sainudiin method [35]. A strict clock was used with a uniform distribution of 1 × 10^−5^–1 × 10^−3^ for the mutation rate of microsatellites, as in previous Bayesian analysis on forest trees [36,37]. Population sizes for Bayesian skyline analysis were set to a rough current estimate of 20,000 trees, calculated starting from an estimate of 88,000 sweet chestnuts for fruit production in Lombardy, as derived from [38]. Trees displaying less than 80% ancestry to a single ancestral gene pool in the *K* = 10 average run were filtered out; the others were sorted by Q values into their respective ancestral populations. The average population-level structure runs were output as a tree (Figure 1), and this was used to construct the monophyletic priors for the Beast analysis. Ten million MCMC chains were run with trees logged every 1000 runs. Tree density (Figure S2) was obtained using DensiTree v.3.0.2, the most probable maximum clade credibility tree was calculated using TreeAnnotator v.2.7.6 [33] after a 20% burn-in period (Figure S3). A stepwise (constant) Bayesian skyline plot was drawn with 2000 bins from the tree root of all individuals (K_root).

## 3. Results

### 3.1. Population Genetic Structure

All the SSR markers used in this study conferred good amplifications in all the varieties analysed, with appropriate polymorphism information content (PIC) values (Table S2). The primers CsCAT41 and CsCAT34 amplified two loci: CsCAT41B has a PIC value equal to 0.558 while CsCAT34B has a value equal to 0.797. The total number of alleles per locus was 267 in 368 samples, with a mean number of 17.8, ranging from 10 alleles in EMCs32 to 31 in CsCAT3.

Given the project’s goal, we started with the assessment of the population structure by means of Structure. The most probable number for *K* was 7, but *K* = 4, *K* = 10 and *K* = 12 also presented sharp peaks (Figure S4). To best explore the whole scenario, we kept these values as reference, and the corresponding bar plots are shown in Figure 1A; however, we opted to use *K* = 10 for demographic analysis as it reflects the historical data more closely. With the exception of Marrone di Limonta (Limonta, for simplicity’s sake), all other varieties showed various degrees of admixture. We also detected a number of trees that behaved the same across different *K* values, showing a distinct origin from the others, and scattered across Limonta, Perledo, Marronessa and Agostana. Given this, we further investigated the origin of the trees with the landowners and other local people, finding that all trees in this group represented another variety, Santa Croce, that was not considered in the original plan of the experiment.

To summarize these findings, we also produced an “average” bar plot at the variety level (Figure 1B). Cluster 1 encompasses the three marron varieties; these are a well-defined cluster with strong differentiation from all other trees. Cluster 2 comprises all other varieties and can be further subdivided into three clusters. Cluster 2a shows an admixed origin, with all varieties displaying a common ancestral gene pool of origin (green). Agostana trees show two main pools of origin (grey and green), Garavina and Bianchit; though admixed, each shows a major ancestral component not present in the other varieties (light green for Garavina and light brown for Bianchit). Cluster 2b also displays an admixed origin and both constituents contain the root “Marron” in their historical variety name and are known to be marron-like chestnut trees. Finally, Cluster 3c encompasses Gulpàt, whose major ancestral contribution is also shared, in part with Bunèla and Vardèe (cyan). The latter two also show a rather large proportion of ancestral pool 1 (red), together with Luina.

### 3.2. Genetic Variability and Differentiation

The distribution of genetic variability among populations was obtained by means of observed and expected heterozygosity, the mean number of alleles per locus, the mean number of effective alleles per locus, the mean number of private alleles per locus, the inbreeding coefficient and the percentage of polymorphic loci, as reported in Table 1.

The studied varieties display remarkably high values of genetic variability, as indicated by values of observed and expected heterozygosity ranging, respectively, from 0.645 (Gulpàt) to 0.821 (Marrone di Limonta) and between 0.553 (Gulpàt) and 0.841 (Marrone di Santa Croce). High levels of genetic variability are also reflected by a heterozygotes excess, and by the mean of F_IS_ equal to −0.078 with a confidence interval ranging from −0.229 to 0.092.

The overall genetic differentiation between populations was significant, with a *F*_ST_ value equal to 0.135 (confidence interval at 95%: 0.114 < *F*_ST_ < 0.155), therefore, ~13.5% of the genetic variation is explained by differences among populations.

The pairwise *F*_ST_ matrix is represented by a heatmap underlying relationships between populations (Table 2), where values are depicted by colours; in green, pairs of populations that are more differentiated and vice versa in red. Values range from the lowest value 0.039 (Marrone di Perledo vs. Santa Croce) to the highest one 0.328 (Marrone di Limonta vs. Bunèla).

**Figure 2:**
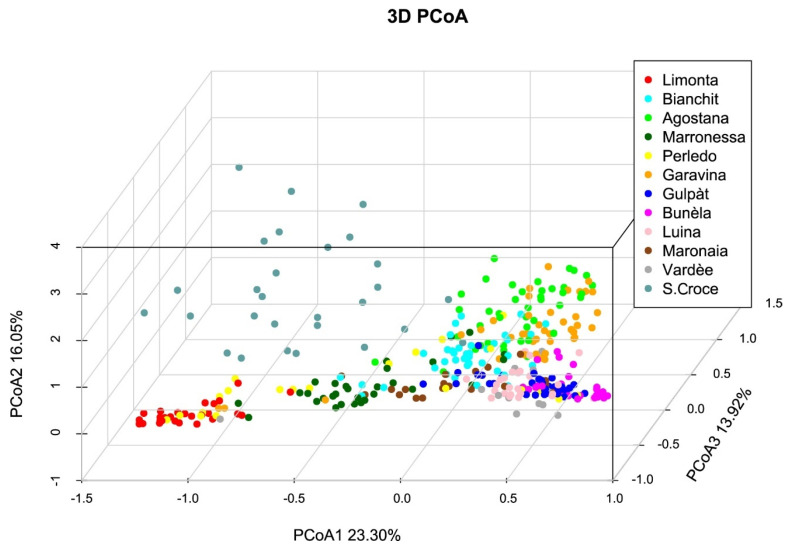
Principal Coordinate Analysis (PCoA) based on the genotypic differences between all individuals. Each colour indicates a different variety, as per the legend.

In the Principal Coordinate Analysis (PCoA), each tree is represented by a dot in a three-dimensional Euclidean space (Figure 2). The three axes represent 53.27% of the total variation. The results are in accordance with the pairwise *F*_ST_ analysis, where the strongest differentiation is exhibited by the Marrone di Limonta vs. Gulpàt and Bunèla varieties.

**Figure 3:**
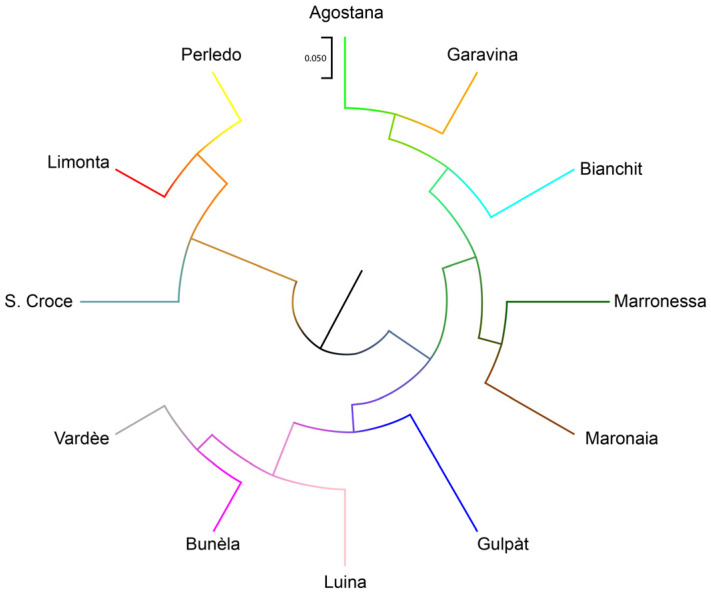
UPGMA tree based on Nei’s genetic distances. Colours are the same as in Figure 2. Scale bar indicates 5% genetic distance.

The Nei genetic pairwise distances between the studied varieties reveal two main clusters, as shown by the UPGMA tree in Figure 3. These are Limonta–Perledo–Santa Croce (Cluster 1) and all other varieties (Cluster 2). Cluster 1 encompasses all marron varieties, while Cluster 2 encompasses all other chestnuts. Cluster 2 further splits into three main clusters: Vardèe–Bunèla–Gulpàt (Cluster 2a), Agostana–Garavina–Bianchit (Cluster 2b) and Marronessa–Maronaia (Cluster 2c).

### 3.3. Demographic Analysis and Divergence

Demography can play a major role in evolutionary processes and help us to understand population dynamics. We performed a Bayesian demographic analysis on our subset of varieties, given the available historical information.

**Figure 4:**
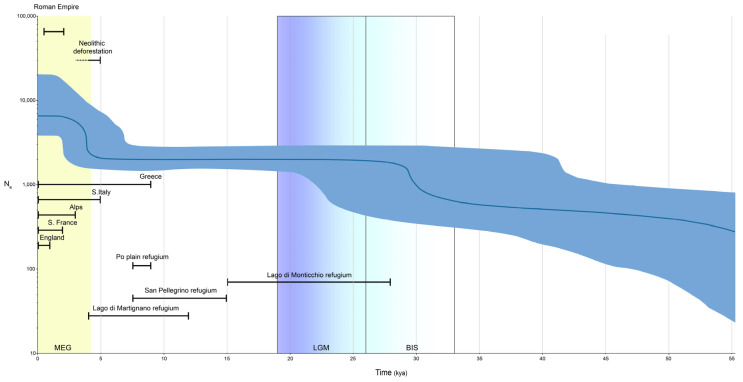
Bayesian skyline plot of chestnut genotypes. The plot considers all 368 trees. The dark blue line indicates the median estimate of the effective population size and the blue shading shows the 95% highest posterior density limits. The time axis is limited to 55 kya, beyond which the curve remains flat. Vertical shaded areas indicate Meghalayan (MEG) in yellow, Last Glacial Maximum (LGM) in blue shades and Beginning of Ice Sheet (BIS) in light blue shading. Bars indicate timespan of given event.

The Bayesian skyline plot (Figure 4) shows the changes in effective population size over time of our chestnut varieties. Two major population growth events can be seen. The first occurred ~30 kya; here the chestnut trees’ effective population size has doubled. Their numbers remained constant throughout the Last Glacial Maximum (LGM) until ~5 kya, when samples reached roughly 14,000 trees, more in line with the current-day estimate of 20,000 trees. Note that BSP N_e_ values appear two-fold smaller than in reality and, given that the software does not account for ploidy, values must, therefore, be doubled [33].

**Figure 5:**
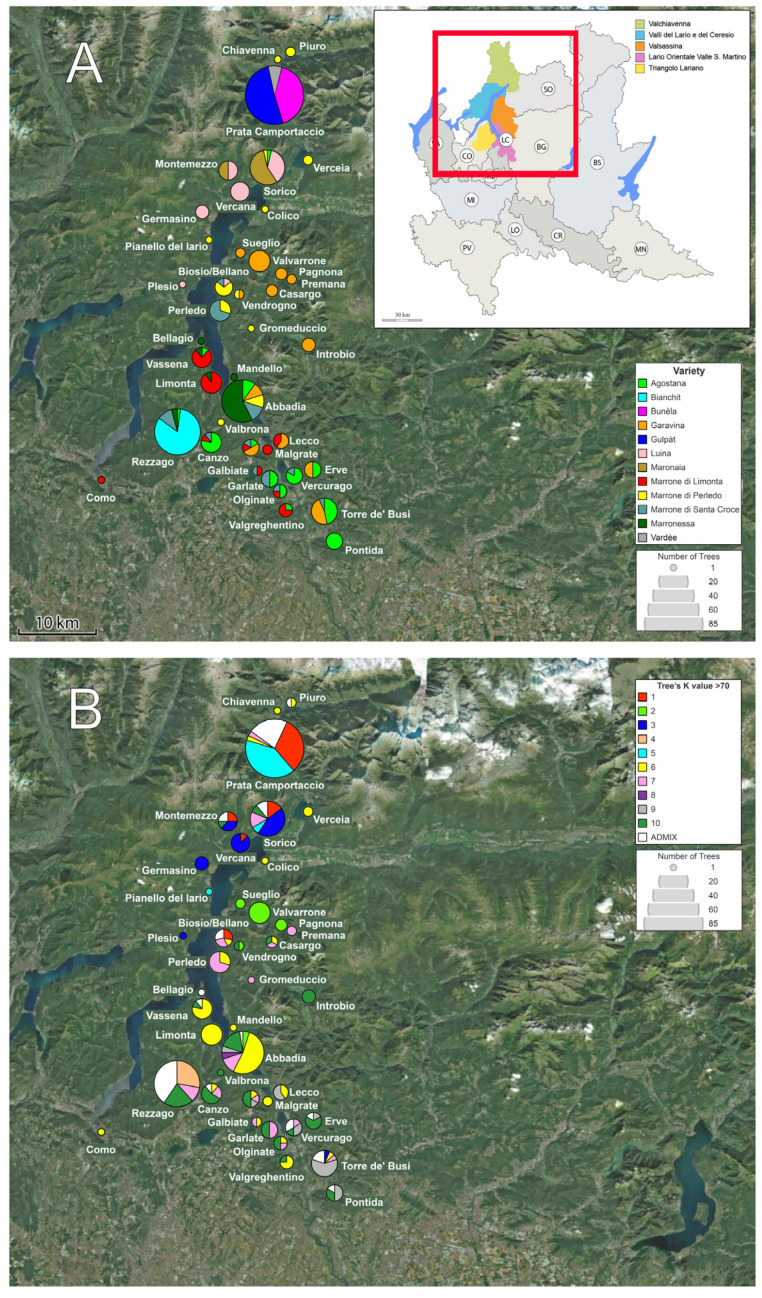
Satellite map of the Lario region, Lombardy, Italy, and sampling municipalities. Circle size represents number of trees. (**A**) Pie charts show the chestnut variety sampled at each location; colours are consistent with Figure 2. (**B**) Pie charts show the proportions of ancestral gene pools of origin for each studied population. The colours match those of the bar chart in Figure 1A,B.

By looking at the divergence times of the Lario trees, we dated the last common ancestor (LarA) to 158 ± 42 kya (Table S3). The first split in the Lario lineage occurred between the admixed populations and all the ancestral pools (*K*-Root), at around 72 ± 1.2 kya. This gave rise to the two major ancestral gene pools, *K*-1, 2, 3, 4, 7 and 9 (N1) and *K*-5, 6, 8 and 10 (N2), which originated, respectively, 71 ± 1.2 kya and 68 ± 1.1 kya. The oldest ancestral pool present to date is that of Marrone di Santa Croce (*K*7), dated to 66 ± 1.1 kya. The other marron variety, Limonta (*K*6), is younger, and dated to 34 ± 0.6 kya. The youngest pools were found to be *K*2 and *K*3, dated to, respectively, 19 ± 0.4 kya and 21 ± 0.6 kya; these are representative today of Garavina and Luina–Maronaia. A representation of the current distribution of the ancestral gene pools can be found in Figure 5.

## 4. Discussion

In this project, we conducted the molecular characterisation of 368 chestnut trees representing eleven different varieties, providing an overview of the distribution of *C. sativa* in the Lario region. The first objective of this study was a genetic assessment of the sweet chestnut in a territory that, albeit covering a small area, still represents the first Italian region where sweet chestnut cultivation was attested to be already taking place at the time of the Roman Empire [9]. This was also done to make an informed inventory of the genetic stocks because the use of vulgar or dialectal names in the identification of trees has created confusion and makes the classification and varietal reorganization complex.

### 4.1. Do Historical Names Reflect Genetics?

It is of importance to renew the interest toward maintaining and propagating chestnut groves for historical variety too, not only for fruit (or timber) production but rather as a cultural legacy to be preserved. In this work, we started comparing “history vs. genetics” not to replace the former with the latter, but to make them complementary in assessing biodiversity. Varietal names were in many cases given by local landowners and indicate some phenological trait, such as “Gulpàt”, dialectal for “volpe”, “fox” in Italian, deriving its name from the colour of the fruit being similar to a fox tail. In other cases, the name was officially stated in civil and ecclesiastical documents going back as far as the XIII century. Genetic analysis revealed that historical and genetic classification are loosely linked. In some instances, such as for Marrone di Limonta, the correspondence between the name given to a tree and its origin as determined by Bayesian analysis is perfect, in other cases, under the same name, trees of different genetic backgrounds are found.

The reasons for the discrepancy can be easily deduced in some cases, such as for “Agostana” (from “Agosto” = “August”), where the name indicates early ripening, a typical quantitative trait under the control of many genetic factors. It can be easily argued that to maintain and propagate this character the farmers grafted early producing trees from different stands onto the rootstocks.

### 4.2. Did We Identify “Heirloom” Varieties?

An “heirloom variety” is a historic cultivar possessing some unique quality; we argue that genetic analysis proved to be useful for varietal identification, even when a formal description and/or other documentation is lacking. This is supported by the purely genetic identification of a variety not present in the original set under study, revealed by the consistent presence at different *K* values of a group of trees with common ancestral origin. After an additional round of interviews with the owners, it was revealed that all these trees also went under another denomination, that is, “Marrone di Santa Croce”, and are characterized by the darker shade of the fruit. In this way, in a sort of double-blind study, our set of SSR markers revealed a hidden variety not fully recognized by the same landowners. This small finding serves, however, to strengthen the need for the assessment of genetic diversity at any ecological scale, from ecosystem to niche, for a correct estimate of biodiversity to be obtained and used for conservation purposes.

### 4.3. Reconstruction of the Varieties’ Diffusion

Other findings of this work can be explained by a comparison of historic and genetic data. Two examples will serve to demonstrate this. The Garavina variety has three main clusters of origin, closely linked to three different, but adjacent, geographic regions. One of the clusters (light green) coincides with the alleged region of origin of the variety. Then, it moved southward, following the main routes for trade and people movement, thus, forming a secondary cluster (dark green) and later a third (grey) population further south. In this case, genetics reconstructed a possible migration route. In a second case, among the trees of the Marrone di Perledo cluster, we can identify three trees belonging to one of the two main clusters of the Agostana variety. All three trees grow between 800 and 900 m a.s.l., at the upper limit of the growing range of sweet chestnut. The Agostana variety is characterised by early ripening, and these trees could benefit from such a characteristic. It may be that some of the markers used in this work are associated with genomic regions controlling ripening.

### 4.4. “Involuntary” Artificial Selection

Sweet chestnut propagation for fruit production has been traditionally performed by grafting selected scions onto suitable rootstocks. This represents a kind of artificial selection, which largely maintained the genetic identity of the original gene pool. The genetic variability assessed by our SSRs (mean number of alleles per locus 17.8) is not different from that found in other studies on Italian populations, being in the range 0.553–0.841, and can be compared with a mean value equal to 0.634 found in another study using a set of 21 SSRs on 55 chestnut trees of the Northern Tuscan Apennines [39]. In fact, the majority of loci have a PIC value > 0.7, which confirms the high discriminating power of these 13 SSRs, and this set of molecular markers has been used in many other studies. In particular, the primer CsCAT3 was one of the most polymorphic and discriminating loci, and this was also found in other studies [15,40].

From this study, it can be concluded that the level of genetic differentiation between these populations around the Lario region is mid–high (*F*_ST_ = 0.135), which is similar to another study, [41], which used ten nuclear microsatellites on four small populations located in the Hyrcanian Forest, and which obtained a value of *F*_ST_ = 0.198. Differentiation appears elevated in such a relatively small geographic area, with this data supporting, again, the idea that the differences between the local sweet chestnut populations have been maintained when the varieties were built by grafting. This is also a tribute to the skill of the first cultivators.

The PCoA results are in agreement with the *F*_ST_ values and the Structure analysis outcome (*K* = 10), displaying that some populations, like the Marrone di Limonta and Bunèla, are more differentiated between each other, revealing a probable variety identity. The traditional names are given on the basis of fruit shape, size, texture and, in the case of marrons, the number of developed fruits in the husk, which is always one. This can be also seen in the UPGMA tree, where marrons and chestnuts are clearly divided, again indicating artificial selection for fruit.

The analysis of population structure clearly revealed for many historical varieties the presence of multiple gene pools of origin. This testifies multiple events of artificial selection at the phenotype level, as can be seen by the same overall fruit phenotype in the case of the two marrons (Limonta and S. Croce) deriving from two ancestral gene pools, and, in the case of early flowering for the Agostana variety, resulting in two gene pools for the same phenotype. The case of Marrone di Perledo, on the other hand, is more convoluted. Its geographical distribution range reaches the most northern point of the studied area, while it shares its distribution in the south with the other two marrons. However, it does not cluster in a homogenous gene pool as individual trees have different origins, mainly belonging to Limonta. This suggests that the more prized Limonta fruit was shared between the different communities and reached the Valchiavenna in the north by crossing the lake; here it took the traditional name of Marrone di Perledo from the town of Perledo, at the crossroads of the northern, eastern and southern mountain communities, though, from the genetic point of view, being part of Limonta (see also Figure 5). Furthermore, we have Bianchit, a variety found linked only to the town of Rezzago in the South (Lariano triangle). The variety was most likely named for the colour of the kernel (Bianchit = bianco, Italian for “white”). Therefore, once again we have a phenotypic trait being selected; however, the population structure analysis revealed three different clusters present within this population: trees with a unique gene pool of origin (*K*4), trees with the Agostana characteristics (*K*10) and an admixed group of Agostana-marron-like ancestry (*K*8, 10), indicating that the selection for a given phenotype, in this case white fruit, resulted in the selection of genetically different trees with similar characteristics. Interestingly, the marron-like varieties partially share an ancestral gene pool, (*K*8), showing that, once again, varieties were selected on the basis of fruit size in a different moment in time. The Marronessa variety, in particular, has a larger proportion of this marron-like ancestry, with the other major component being Limonta, hinting at a hybrid origin in the region where they are both present. It is worth noting that Marronessa produces three fruits per husk, indicating its “chestnut” origin; however, in drought years it is known to produce just one fruit of greater dimensions, like a marron does.

Finally, by studying the demography of the species, we can see how events that shaped the population size and diffusion can be detected. The effective population size did not change much until the beginning of the LGM, when the number of trees started rising. This could initially seem counterintuitive, but, comparing these dates with previously published data, we can see how this species found four main refugia in Italy [5,6,9] and, thus, found a niche to thrive in during the harsh, cold conditions. Furthermore, these refugia extended temporarily from the LGM into the MEG, roughly following a south-to-north direction, roughly reaching our studied area ~8 kya (Po plain refugium), as shown by pollen records. A second demographic event can be seen ~5 kya with another rapid increment in population size; this time period corresponds to the Neolithic in Europe. The increment in population size has, therefore, been linked to decreased competition with other species due to anthropogenic deforestation [42]. Another certified population increase occurred ~2 kya, when the Romans brought the tree from southern Italy across the Alps, into Southern France and later into England, as demonstrated by isozyme data [42]. Unfortunately, given the small sampling area, this last increase can only be indirectly inferred by the large confidence interval observed in our study. The use of uniparental markers such as chloroplast or mitochondrial DNA could help further shed light on the species dispersion through the Roman empire.

### 4.5. Final Remarks

Besides its traditional uses for fruit (“bread-tree”) and timber production, from a modern point of view, a chestnut grove provides to the community very important services, like an ecological contribution with the capture and sequestration of CO_2_ and containment of hydrogeological instability and forest fires, but also protects biodiversity, in particular the fauna which find their ideal habitat in the Castanetum. To preserve the existing chestnut heritage, it is important to plan recovery strategies for the varieties at risk of erosion, for monumental specimens and for mother plants and their germplasm. Rapid shifts in climate patterns and the continued waves of pathogens can affect chestnut productivity and health, necessitating adaptive management strategies. Within this framework, our work represents a likely model for probing biodiversity with the goal of maintaining both genetic diversity and cultural memes, both in Lario and in other regions.

## 5. Conclusions

In this work, the molecular and demographic analysis of historical varieties allowed the characterization of the genetic makeup and structure of sweet chestnut trees around eastern Lario.

The main findings are that, based on the assessment of genetic variability and differentiation, some varieties appear clearly differentiated, namely those, such as Marrone di Limonta, whose origin can be traced back with more certainty. Then, Bayesian analysis of the population structure allowed us to pinpoint relationships among the studied varieties by determining multiple gene pools of origin, which can also be seen as different selection events for desirable characteristics of the fruit, such as sweetness, texture, and others. In some cases, we also inferred possible diffusion routes for the chestnut trees. Last, Bayesian demographic reconstruction was used to date ancestral lineages, and the inferred dates fit well with the documented history of sweet chestnut in the Italian peninsula.

All these results taken together should, in our opinion, prompt the study of the chestnut trees to preserve biodiversity and to eventually identify new varieties, with the consequent possibility of recognizing the PGI (Protected Geographical Indication) and PDO (Protected Designation of Origin) marks that qualify the products, giving added value.

## Figures and Tables

**Table 1:** Expected (*H_e_*) and observed (*H_o_*) heterozygosity over loci, mean number of alleles per locus (Na), mean number of effective alleles per locus (Nea), mean number of private alleles per locus (Npa), inbreeding coefficient (*F*_IS_) and percentage of polymorphic loci (%pl) for each studied variety.

	*H_e_*	*H_o_*	Na	Nea	Npa	*F*_IS_	% pl
Marrone di Limonta	0.558	0.821	4.000	2.332	0.00	−0.471	100.00
Bianchit	0.698	0.696	8.267	4.019	0.333	0.003	100.00
Agostana	0.720	0.724	9.600	4.200	0.267	−0.005	100.00
Marronessa	0.671	0.815	7.733	3.264	0.200	−0.215	100.00
Marrone di Perledo	0.699	0.727	6.600	3.567	0.067	−0.040	100.00
Garavina	0.694	0.673	9.267	3.684	0.533	0.030	100.00
Gulpàt	0.553	0.645	6.933	2.422	0.467	−0.166	100.00
Bunèla	0.585	0.695	6.067	2.561	0.267	−0.188	100.00
Luina	0.595	0.694	5.267	2.600	0.133	−0.166	100.00
Maronaia	0.630	0.714	6.733	3.180	0.267	−0.133	93.33
Vardèe	0.648	0.752	4.867	3.011	0.133	−0.160	100.00
Marrone di Santa Croce	0.841	0.720	12.000	6.907	1.600	0.144	100.00

**Table 2:** Matrix of *F*_ST_ values between variety pairs; the closest are in green and the farthest are in red. All the values are statically significant (*p* ≤ 0.05).

	Bianchit	Agostana	Marronessa	Perledo	Garavina	Gulpàt	Bunèla	Luina	Maronaia	Vardèe	S. Croce
**Limonta**	0.181	0.211	0.139	0.064	0.212	0.319	0.328	0.286	0.216	0.269	0.111
**Bianchit**	-	0.066	0.079	0.066	0.074	0.110	0.156	0.116	0.087	0.115	0.073
**Agostana**	-	-	0.110	0.083	0.051	0.182	0.162	0.124	0.088	0.123	0.082
**Marronessa**	-	-	-	0.054	0.103	0.180	0.183	0.142	0.083	0.110	0.073
**Perledo**	-	-	-	-	0.095	0.190	0.168	0.113	0.073	0.116	0.039
**Garavina**	-	-	-	-	-	0.157	0.139	0.125	0.107	0.097	0.097
**Gulpàt**	-	-	-	-	-	-	0.157	0.162	0.182	0.150	0.179
**Bunèla**	-	-	-	-	-	-	-	0.080	0.162	0.052	0.174
**Luina**	-	-	-	-	-	-	-	-	0.085	0.079	0.125
**Maronaia**	-	-	-	-	-	-	-	-	-	0.128	0.098
**Vardèe**	-	-	-	-	-	-	-	-	-	-	0.104

## Data Availability

The original contributions presented in the study are included in the article/Supplementary Material, further inquiries can be directed to the corresponding author/s.

## Supplementary Materials

The following supporting information can be downloaded at: https://www.mdpi.com/article/10.3390/genes15070866/s1. Figure S1: Lombardy mountain communities (CMs) involved in the project; Figure S2: Genetic structure bar plot of the studied samples as inferred by Bayesian clustering for K = 4, 7, 10 and 12; Figure S3A: Average tree for *K* = 10, N1 and N2 refer to the ancestral roots of the two main clusters; Figure S3B: Box and whisker plot of main cluster and node divergence times; Figure S3C: Density distribution of the best 10,000 trees (in green) calculated via Bayesian analysis using Beast; Figure S4: Graphical Δ*K* representation of the estimated probability of data for each K-value from K = 1 to K = 15; Table S1: Names, position and number of the ten local varieties; Table S2: Features of the microsatellites used in this study and Table S3: Bayesian age estimates of main nodes and ancestral pools.
