# Supplementary material for: Genetic Insights into the Historical Attribution of Variety Names of Sweet Chestnut (Castanea sativa Mill.) in Northern Italy
Source: Genes (Basel). 2024 Jul 1;15(7):866. doi: 10.3390/genes15070866 (PMC11276188; doi:10.3390/genes15070866)
Supplement: Supplementary file 1 [file genes-15-00866-s001.zip › genes-3072904-supplementary.pdf]

Supplementary Material

Genetic insights into the historical attribution of variety names of sweet chestnut (*Castanea sativa* Mill.) in Northern Italy

Marta Cavallini[1,†], Gianluca Lombardo[1,†], Claudio Cantini[2], Mauro Gerosa[3], Giorgio Binelli[1,*]

[1] Department of Biotechnology and Life Sciences (DBSV), University of Insubria, 21100 Varese, Italy
[2] Institute of Bioeconomy (IBE), Consiglio Nazionale Ricerche (CNR), 58022 Follonica, Italy
[3] Associazione Castanicoltori Lario Orientale, 23851 Galbiate, Italy

* Correspondence: giorgio.binelli@uninsubria.com
† These authors contributed equally to this work.

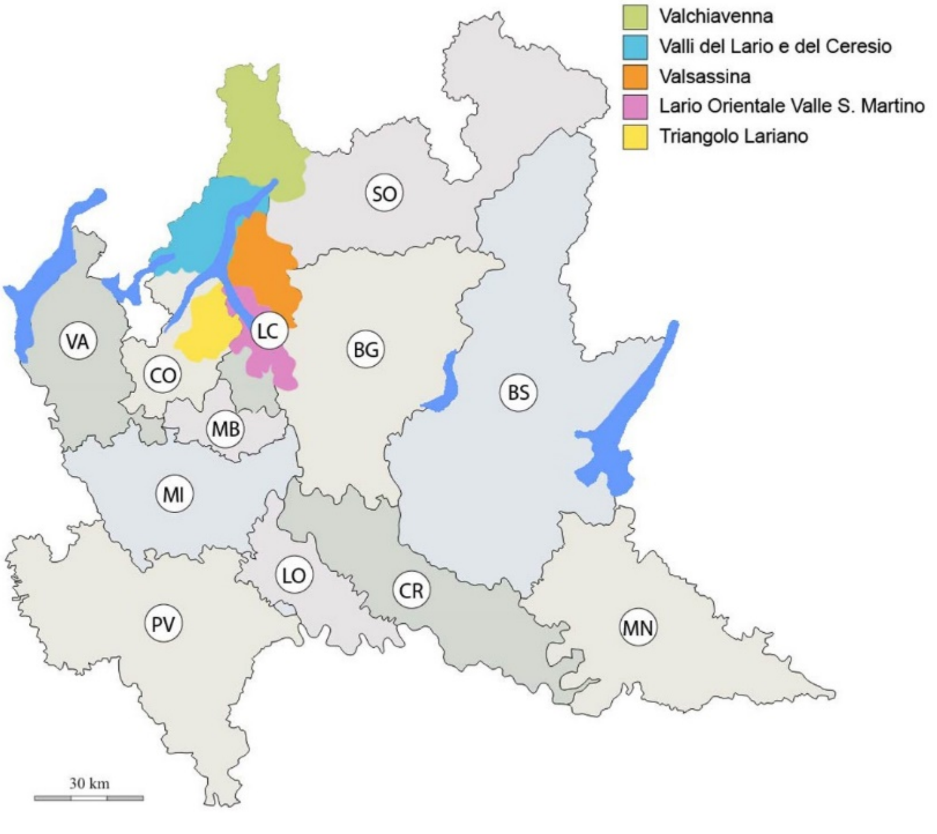

Supplementary Figure S1. Lombardy mountain communities (CMs) involved in the project.

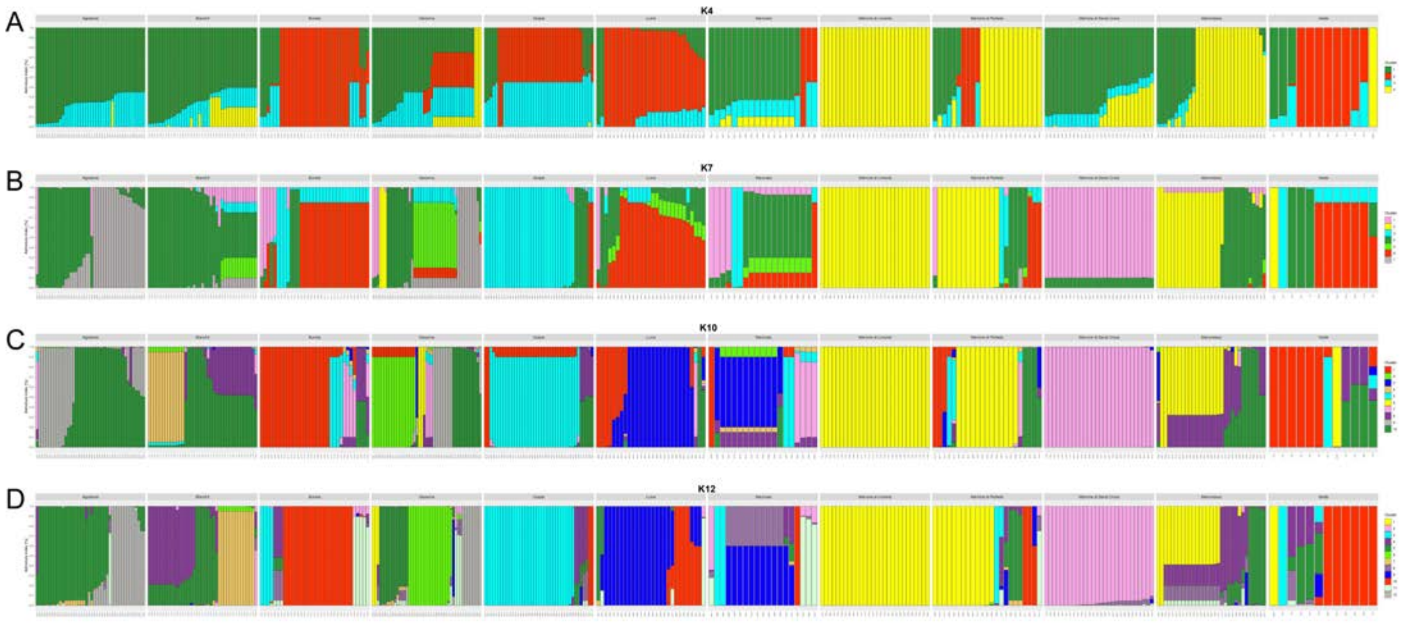

Supplementary Figure S2. Genetic structure bar plot of the studied samples as inferred by Bayesian clustering for K = 4, 7, 10 and 12. Each vertical bar represents a single tree and its proportion of membership to a genetic pool is indicated by the different colours.

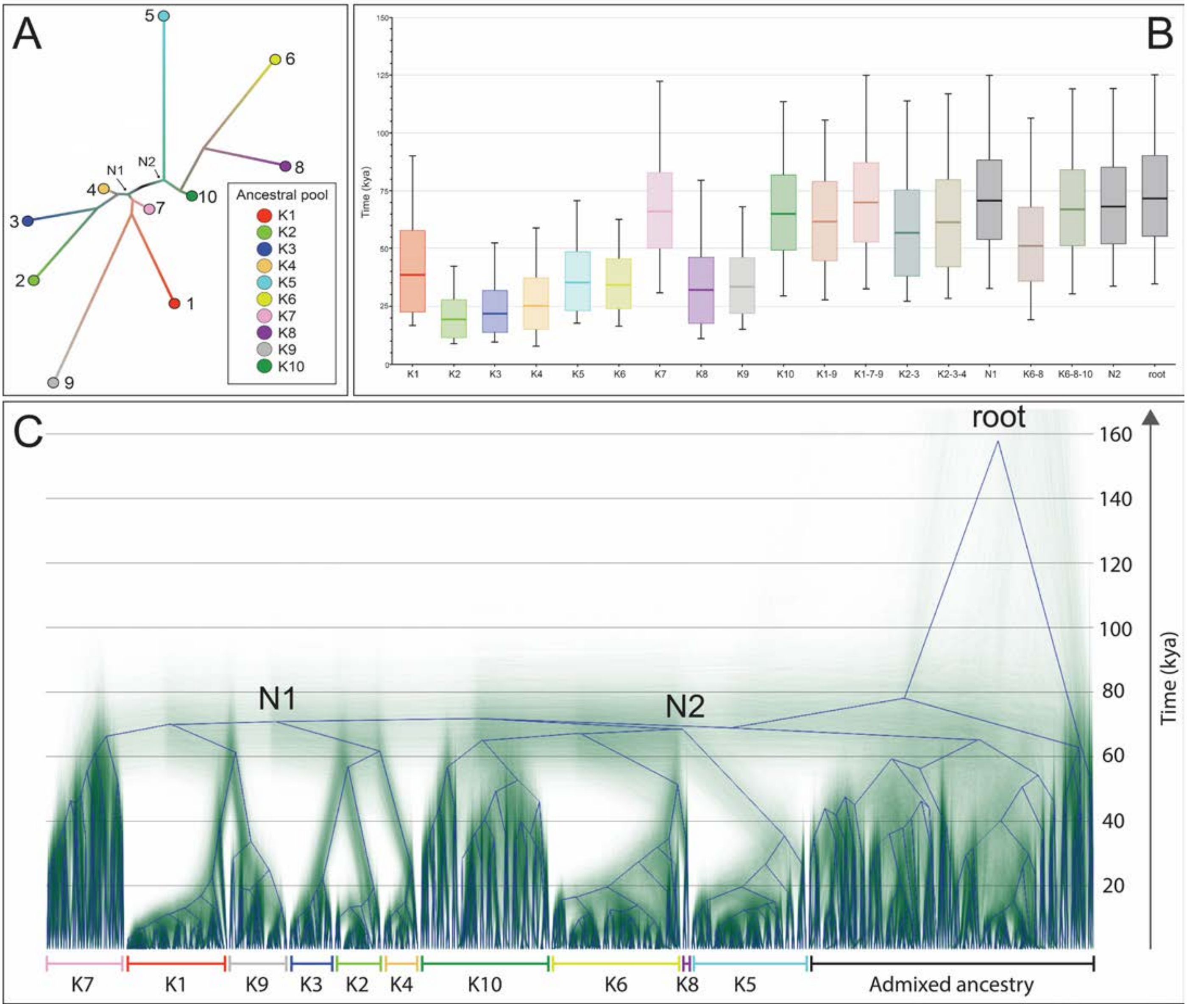

Supplementary Figure S3. A. Average tree for *K*=10, N1 and N2 refer to the ancestral roots of the two main clusters. Colours are the same as all *K*=10 Structure results. B. Box and whisker plot of main cluster and node divergence times. Solid lines inside box represent the median, coloured boxes are the 95% credible interval and the whiskers are the value ranges. C. Density distribution of the best 10,000 trees (in green) calculated via Bayesian analysis using Beast. The solid blue line is the consensus tree given 20% burn-in. Coloured bars below clades represent the different ancestral pool of origin.

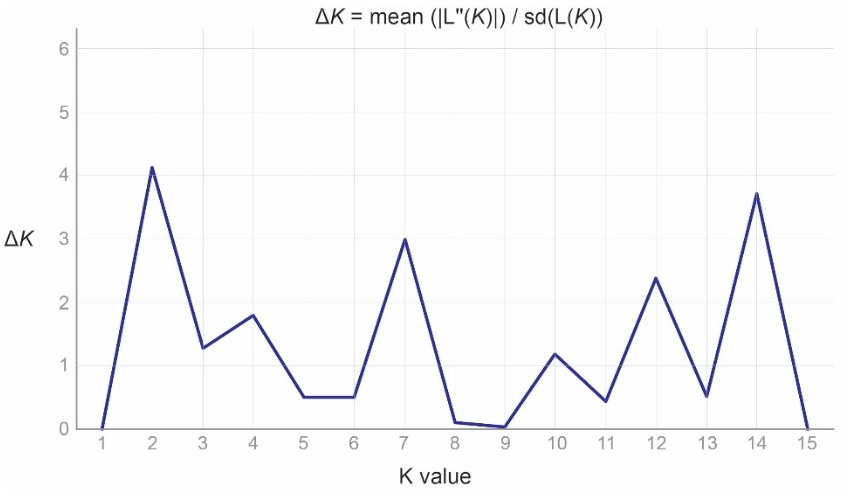

Supplementary Figure S4. Graphical Δ*K* representation of the estimated probability of data for each K-value from K = 1 to K = 15. No-Admixture model with LOCPRIOR, 100,000 MCMC iterations and a 10,000 iterations burn-in period.

Varities	Mountain communities	N° samples
Marrone di Limonta	Triangolo Lariano	37
Bianchit	Triangolo Lariano	40
Agostana	Lario Orientale e Valle San Martino	46
Marronessa	Lario Orientale e Valle San Martino	36
Perledo	Valsassina	32
Garavina	Valsassina	45
Gulpàt	Valchiavenna	40
Bunèla	Valchiavenna	33
Luina	Valli del Lario e del Ceresio	28
Maronaia	Valli del Lario e del Ceresio	19
Vardèe	Valchiavenna	12

Supplementary Table S1. Names, position and number of the ten local varieties.

Supplementary Table S2. Features of the microsatellites used in this study. Note that only Forward primers were labelled with fluorescent dyes. PIC = polymorphism information content. Primers were used in multiplexed PCRs as indicated by the Mix number of the first column.

**Multiplex**	**SSR Name**		**Sequence (5′ → 3′)**	**Repeat motif**	**Allele sizes**	**T_a (°C)**	**PIC**
**Mix 1**	CsCAT3 – TAMRA	F:	CACTATTTTATCATGGACGG	$(AG)_{20}$	182-252	50	0.846
		R:	CGAATTGAGAGTTCATACTC				
	CsCAT6 – HEX	F:	AGTGCTCGTGGTCAGTGAG	$(AC)_{24}AT(AC)_{4}$	152-196	50	0.751
		R:	CAACTCTGCATGATAAC				
	CsCAT14 – ROX	F:	CGAGGTTGTTGTTCATCATTAC	$(CA)_{22}$	131-163	58	0.680
		R:	GATCTCAAGTCAAAAGGTGTC				
	CsCAT16 – FAM	F:	CTCCTTGACTTTGAAGTTGC	$(TC)_{20}$	120-166	50	0.711
		R:	CTGATCGAGAGTAATAAAG				
**Mix 2**	CsCAT1 - HEX	F:	GAGAATGCCCACTTTTGCA	$(TG)_{5}TA(TG)_{24}$	184-226	50	0.677
		R:	GCTCCCTTATGGTCTCG				
	CsCAT2 - TAMRA	F:	GTAACTTGAAGCAGTGTGAAC	$(AG)_{16}$	202-246	55	0.853
		R:	CGCATCATAGTGAGTGACAG				
	CsCAT17 – ROX	F:	TTGGCTATACTTGTTCTGCAAG	$(CA)_{19}A(CA)_{2}AA(CA)_{3}$	133-163	58	0.782
		R:	GCCCCATGTTTTCTTCCATGG				
	CsCAT34 – FAM	F:	TGAGCAAGGATGGATGATGAG	$(GT)_{23}$	140-166	50	0.569
		R:	GGTGGTCATCATGACTGCATC				
**Mix 3**	CsCAT41 - ROX	F:	AAGTCAGCAACACCATATGC	$(AG)_{20}$	199-241	53	0.795
		R:	CCCACTGTTCATGAGTTTCT				
	EMCs25 – FAM	F:	ATGGGAAAATGGGTAAAGCAGTAA	$(GA)_{12}$	134-168	54	0.674
		R:	AACCGGAGATAGGATTGAACAGAA				
	EMCs38 – HEX	F:	TTTCCCTATTTCTAGTTTGTGATG	$(AG)_{31}$	220-270	50	0.890
		R:	ATGGCGCTTTGGATGAAC				
**Mix 4**	EMCs22 – FAM	F:	GTGCCTCTGTATGCATGGTAAGC	$(GA)_{19}$	124-160	57	0.836
		R:	CCAGGTTTAAGAAAGCAAGCATAAC				
	EMCs32 – ROX	F:	TTCCACACTTATCTCTTAACCCAAAAA	$(AG)_{18}$	88-160	60	0.487
		R:	CTCCGGTACGGTATTGACTTCCTT				

Node/ancestral pool	N° plants	Divergence times (kya)	
		Mean T	Mean ΔT
Root	368	158.4	41.9
> K-Root	368	71.7	1.2
>> N1	131	70.6	1.2
>>> 1-7-9	85	69.9	1.2
>>>> 1-9	57	61.4	0.9
>>>>> 1	36	38.5	0.7
>>>>> 9	21	33.4	0.9
>>>> 7	28	66.0	1.1
>>> 2-3-4	46	61.5	1.0
>>>> 2-3	33	56.7	0.9
>>>>> 2	17	19.3	0.4
>>>>> 3	16	21.9	0.6
>>>> 4	13	25.2	0.5
>> N2	136	68.4	1.1
>>> 6-8-10	95	67.0	1.1
>>>> 6-8	49	51.3	0.8
>>>>> 6	46	34.4	0.6
>>>>> 8	3	31.8	0.6
>>>> 10	46	65.0	1.0
>>> 5	41	35.2	0.6

Supplementary Table S3. Bayesian age estimates of main nodes and ancestral pools. Estimates are based on the Bayesian phylogeny of Fig. trees. Number of trees and standard error are also reported.
